# Strain surveillance during chemotherapy to improve cardiovascular outcomes: the SUCCOUR-MRI trial

**DOI:** 10.1093/eurheartj/ehae574

**Published:** 2024-09-01

**Authors:** Thomas H Marwick, Elizabeth Dewar, Mark Nolan, Mitra Shirazi, Peter Dias, Leah Wright, Ben Fitzgerald, Leighton Kearney, Piyush Srivastava, John Atherton, Kazuaki Negishi, Aaron L Sverdlov, Sudhir Wahi, James Otton, Joseph Selvanayagam, Liza Thomas, Paaladinesh Thavendiranathan

**Affiliations:** Imaging Research, Baker Heart and Diabetes Institute, Melbourne, VIC, Australia; Cardiovascular Imaging, Menzies Institute for Medical Research, University of Tasmania, Hobart, Australia; Imaging Research, Baker Heart and Diabetes Institute, Melbourne, VIC, Australia; Imaging Research, Baker Heart and Diabetes Institute, Melbourne, VIC, Australia; Cardio-Oncology Section, Peter MacCallum Cancer Centre, Melbourne, VIC, Australia; Department of Cardiology, Royal Adelaide Hospital, Adelaide, SA, Australia; Advara Heart Care, Murdoch, WA, Australia; Imaging Research, Baker Heart and Diabetes Institute, Melbourne, VIC, Australia; Advara Heart Care, Brisbane, QLD, Australia; Advara Heart Care, Melbourne, VIC, Australia; Advara Heart Care, Melbourne, VIC, Australia; University of Queensland Faculty of Medicine, Royal Brisbane and Women’s Hospital, Brisbane, QLD, Australia; Cardiology Department, Nepean Hospital, Kingswood, NSW, Australia; Nepean Clinical School, University of Sydney, Kingswood, NSW, Australia; Newcastle Centre of Excellence in Cardio-Oncology, The University of Newcastle, Hunter Medical Research Institute, Calvary Mater Newcastle, Hunter New England Health, Newcastle, NSW, Australia; Princess Alexandra Hospital, Brisbane, Australia; Liverpool Hospital, Liverpool, NSW, Australia; Flinders University, Adelaide, SA, Australia; Westmead Clinical School, University of Sydney and University of New South Wales, Sydney, NSW, Australia; Peter Munk Cardiac Centre, Toronto General Hospital, Toronto, Ontario, Canada

**Keywords:** Cancer therapy-related cardiac dysfunction, Left ventricular ejection fraction, Global longitudinal strain

## Abstract

**Background and Aims:**

The detection of cancer therapy-related cardiac dysfunction (CTRCD) by reduction of left ventricular ejection fraction (LVEF) during chemotherapy usually triggers the initiation of cardioprotective therapy. This study addressed whether the same approach should be applied to patients with worsening of global longitudinal strain (GLS) without attaining thresholds of LVEF.

**Methods:**

Strain surveillance during chemotherapy for improving cardiovascular outcomes (SUCCOUR-MRI) was a prospective multicentre randomized controlled trial involving 14 sites. Of 355 patients receiving anthracyclines with normal baseline LVEF, 333 patients (age 59 ± 13 years, 79% women) with at least one other CTRCD risk factor, able to undergo magnetic resonance imaging (MRI), GLS, and three-dimensional echocardiography were tracked over 12 months. A total of 105 patients (age 59 ± 13 years, 75% women, 69% breast cancer) developing GLS-CTRCD (>12% relative reduction of GLS without a change in LVEF) were randomized to cardioprotection with neurohormonal antagonists vs. usual care. The primary endpoint was 12-month change in MRI-LVEF; the secondary endpoint was MRI-LVEF-defined CTRCD.

**Results:**

During follow-up, two patients died, and two developed heart failure. Most patients were randomized at 3 months (62%). Median doses of angiotensin inhibition/blockade and beta-blockade were 75% and 50% of respective targets; 21 (43%) had side-effects attributed to cardioprotection. Due to a smaller LVEF change from baseline with cardioprotection than usual care (−2.5 ± 5.4% vs. −5.6 ± 5.9%, *P* = .009), follow-up LVEF was higher after cardioprotection (59 ± 5% vs. 55 ± 6%, *P* < .0001). After adjustment for baseline LVEF, the mean (95% confidence interval) difference in the change in LVEF between the two groups was −3.6% (−1.8% to −5.5%, *P* < .001). After cardioprotection, 1/49 patients developed 12-month LVEF-CTRCD, compared to 6/56 in usual care (*P* = .075). Global longitudinal strain improved at 3 months post-randomization in the cardioprotection group, with little change with usual care.

**Conclusions:**

In patients with isolated GLS reduction after anthracyclines, cardioprotection is associated with better preservation of 12-month MRI-LVEF compared with usual care.

## Structured Graphical Abstract

**Structured Graphical Abstract:**
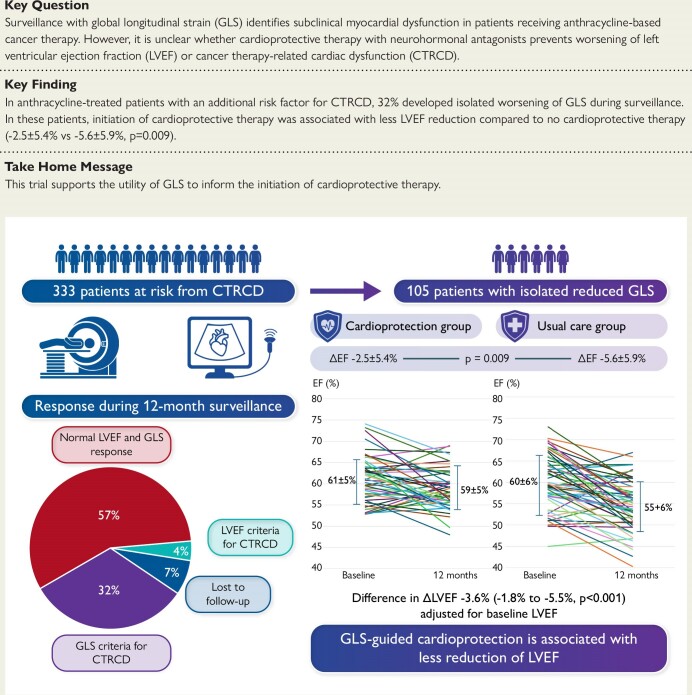
From 355 patients with normal left ventricular function undergoing anthracycline therapy, 333 with additional risk factors for cancer therapy-related cardiac dysfunction (CTRCD) were able to undergo baseline and magnetic resonance imaging and echocardiography. Of these, 105 developed isolated impairment of global longitudinal strain (GLS), and were randomized to cardioprotection with neurohormonal inhibition or usual care. Patients taking cardioprotection experienced a smaller change in left ventricular ejection fraction (LVEF) over 12 months. The baseline-corrected mean difference in LVEF was −3.6%. Normal LVEF and GLS = not meeting an LVEF-CTRCD criteria or a GLS change >12%; GLS only = an isolated >12% relative reduction in GLS; Lost = patients lost to follow-up. EF = patients who met the LVEF-CTRCD criteria.

**See the editorial comment for this article ‘From setbacks to success: building a promising path for strain-guided cardioprotection during anthracycline treatment’, by A. Barac *et al*., https://doi.org/10.1093/eurheartj/ehae598.**

## Introduction

Anthracyclines remain widely used in the management of several malignancies,^1^ and despite limiting anthracycline doses, cancer therapy-related cardiac dysfunction (CTRCD) remains a significant problem. In addition, the use of trastuzumab and many other anti-cancer agents may also produce cardiac impairment.^2^ Thus, cardiovascular disease remains an important concern for cancer survivors, with early stage breast cancer patients more likely to die from heart disease than cancer.^3^ The reported incidences of left ventricular (LV) dysfunction (5%–15%) and heart failure (HF, 0%–5%) in observational studies^4^ are exceeded by epidemiologic studies showing respective 3-year incidences ranging from 18%–42%.^5^

Left ventricular ejection fraction (LVEF) is ubiquitous in surveillance for CTRCD. Recent cardio-oncology guidelines propose the identification of moderate CTRCD based on reduction of LVEF to 40%–49% with ≥10% absolute change in LVEF (or <10% change in the presence of relative global longitudinal strain [GLS] change >15%).^6^ However, 2-dimensional (2D) echocardiography is most widely used for surveillance, and 2D LVEF presents a number of challenges related to image quality, assumption of LV geometry, and expertise.^7^ Because decisions are made from sequential testing, test–retest reliability is important, and absolute LVEF changes of <10% with 2D echocardiography,^8^ and <5% with 3-dimensional (3D echocardiography)^9^ may not be meaningful. Cardiac magnetic resonance (MRI) provides consistently excellent image quality, and the ability to detect changes of ≤4%,^10^ but its cost and availability for repeated testing remain a challenge. There have been extensive studies of 2D GLS for CTRCD surveillance,^11^ but there remains lack of consensus as to whether isolated reduction of GLS (without a meaningful change of LVEF) should warrant cardioprotection. The current cardio-oncology guidelines characterize GLS change in the presence of normal LVEF as mild CTRCD, with cardioprotection given a Class IIa indication. The strain surveillance during chemotherapy for improving cardiovascular outcomes (SUCCOUR-MRI) trial tested whether the use of cardioprotective therapy in patients with a significant isolated reduction in GLS (>12% relative change) limits the decrement in MRI-measured LVEF and development of CTRCD at 12 months.

## Methods

### Design

This international, multicentre trial used a prospective randomized open-blinded endpoint (PROBE) design to study patients at risk of CTRCD, undergoing echocardiographic surveillance of LV function during chemotherapy. The trial arose from this research group’s experience with the original SUCCOUR trial, but is a completely independent trial, with intentional differences in design (SUCCOUR randomized patients to LVEF vs. GLS-guided cardioprotection in all patients, SUCCOUR-MRI randomized those with abnormal GLS response to cardioprotection [neurohormonal blockade] vs. no therapy [usual care]), with different EF modalities for defining EF (3D echocardiography vs. MRI) and differences in risk profile (*Table 1*).

**Table 1:** Baseline and follow-up characteristics of SUCCOUR-MRI patients, compared with the patients from the original SUCCOUR trial

	SUCCOUR-MRI (*n* = 333)	SUCCOUR (*n* = 307)	*P*
Age, years	59 ± 13	53 ± 10	<.0001
Female, *n* (%)	262 (79%)	288 (94%)	<.0001
Diabetes, *n* (%)	55 (17%)	39 (9.1%)	.17
Hypertension, *n* (%)	128 (38%)	89 (27.9%)	.01
Dyslipidemia, *n* (%)	102 (31%)	64 (17.5%)	.005
Smoking^a^, *n* (%)	125 (38%)	90 (29.9%)	.03
Other cardiovascular disease, *n* (%)^b^	52 (16%)	30 (10.4%)	.03
Beta blocker, *n* (%)	11 (3%)	15 (5.2%)	.31
ACE inhibitor or ARB, *n* (%)	89 (27%)	43 (13.6%)	<.0001
Statin, *n* (%)	96 (29%)	42 (9.7%)	<.0001
Systolic blood pressure, mmHg	128 ± 16	128 ± 24	.70
Diastolic blood pressure, mmHg	75 ± 10	76 ± 10	.59
Heart rate, beats/min	77 ± 14	77 ± 12	.62
Cancer history			
Breast cancer	251 (75%)	278 (91%)	<.0001
Lymphoma or leukaemia	77 (23%)	29 (9%)	
Other (sarcoma)	2 (1%)		
Baseline measurement			
3D LVEF, %	61 ± 6	58 ± 6	.0001
GLS,%	−19.8 ± 2.5	−20.5 ± 2.4	.585
MRI-LVEF, %	62 ± 9		
CTRCD over 12 months (pre-cardioprotection)			
GLS-defined	105 (32%)	44/154 (29%)	.51
Echo-EF-defined	12 (4%)^c^	27 (8.7%)	.01

Continuous data are presented as median (Q1–Q3) or as mean ± standard deviation.

^a^Current or prior smoking.

^b^Patients with coronary artery disease, mild valvular heart disease, and other non-major cardiovascular disease (CVD) (heart failure and moderate and greater valve disease excluded in both studies).

^c^Note that CTRCD was defined as LVEF fall >10% to <50% in SUCCOUR-MRI and >10% fall to <55% in SUCCOUR.

Patients with normal baseline echocardiographic LVEF (>50%), at risk for CTRCD due to anthracycline therapy in combination with another risk for CTRCD or completed anthracycline therapy and planned for ongoing potentially cardiotoxic treatment (e.g. trastuzumab or tyrosine kinase inhibitors), were eligible for the study (*Table 2*). Participants were recruited from 14 Australian and Canadian sites (see Supplementary data online, *Appendix Table S1*), with the co-ordinating site at the Baker Institute, Melbourne, which was responsible for data management and the core imaging laboratory for primary endpoint determination.

**Table 2:** Inclusion and exclusion criteria

Inclusion criteria
1. Patients at increased risk of cardiotoxicity
**Either** pending/active anthracycline therapy WITH one of the following (not necessarily concurrently) with Other potentially cardiotoxic chemotherapy (e.g. trastuzumab, tyrosine kinase inhibitors) OR Cumulative anthracycline doses >450 mg/m^2^ OR Current chest radiotherapy (left sided) OR Past anthracycline (any dose) or chest radiotherapy OR Increased risk of HF (age >65y, Type 2 diabetes mellitus, hypertension, previous cardiac injury, e.g. myocardial infarction)
**Or** patients who have completed anthracycline treatment but will continue to be treated with other cardiotoxic treatments (e.g. trastuzumab, tyrosine kinase inhibitors) during surveillance
2. Live within a geographically accessible area for follow-up
3. Are able and willing to provide written informed consent to participate in the study (this includes the ability to communicate fluently with the investigator and that the patient is mentally competent)
**Exclusion criteria**
Unable to provide written informed consent to participate in this study
Oncologic (or other) life expectancy <12 months or any other medical condition (including pregnancy) that results in the belief (deemed by the Chief Investigators) that it is not appropriate for the patient to participate in this trial
Participating in another clinical research trial where randomized treatment would be unacceptable
Pre-existing cardiovascular disease History of previous heart failure (baseline NYHA >2) Baseline 3D-echocardiographic left ventricular ejection fraction <50% Valvular stenosis or regurgitation of >moderate severity
Inability to acquire interpretable images (identified from baseline echo)
Features interfering with potential neurohormonal inhibition Systolic BP <110 mmHg Pulse <60/min Contraindications/intolerance to beta-blockers or ACE inhibitors Existing therapy with both beta-blockers and ACE inhibitors
Contraindications to MRI, end-stage renal disease (dialysis, eGFR <25)

eGFR, estimated glomerular filtration rate.

### Clinical evaluation

Baseline measures included socio-demographic factors, cardiovascular risk factors, cancer history, comorbidities, medications, and blood pressure by cuff sphygmomanometer. Clinical review was repeated at 3-month intervals through 12 months. Safety evaluations were performed at these 3-month visits by recording adverse events (AEs), serious adverse events (SAEs), and by monitoring laboratory parameters, physical examinations, ECGs, and vital signs (see Supplementary data online, *Appendix Table S2*). Standardized data were acquired using online Case Report Forms, with archiving through a secure web-based database (Redcap). The study monitors at the data co-ordinating centre verified these data for completeness. The same database was used for randomization.

### Echocardiography

Patients underwent standard echocardiograms^12^ at baseline and at 3-month intervals during follow-up, with standard assessment of systolic function^13,14^ (see Supplementary data online, *Appendix Figure S1*). Left ventricular ejection fraction was measured preferentially with 3D echocardiography, with offline measurement of LV volumes and LVEF (EchoPAC, 4D Auto LVQ)—2D echocardiography was only used when 3D LVEF measurement was not possible for technical reasons (*n* = 2). Myocardial peak systolic GLS assessment was obtained from three LV-focused apical views, obtained at a frame rate of at least 70 frames/s, and saved digitally in raw data format. The three apical views were analysed at each site, using semi-automated speckle-tracking (EchoPAC, GE Medical Systems, Milwaukee, WI).^15^ All measures were made in a blinded fashion by a single observer at each site. As in the initial SUCCOUR trial, a standardized training and accreditation programme was co-ordinated through the core laboratory to ensure that each laboratory undertaking ‘point-of-care’ GLS measurement was obtaining accurate images.^16^ This process has provided a very good concordance of GLS results.^17^ Baseline and follow-up images were sent to the core laboratory and randomly sampled for quality control.

At each 3-month visit, it was anticipated that participants would fall into three groups, based on their change from baseline. Most were expected to show *no change*—normal LVEF (≥50%) with no relative reduction of GLS (i.e. <12%). Some were expected to develop *overt dysfunction*—evidenced by abnormal LVEF (<50%) with absolute reduction of echo LVEF (≥5% if symptomatic, ≥ 10% asymptomatic). The remainder were expected to show *subclinical dysfunction* (≥12% relative reduction of GLS compared to baseline in the absence of overt dysfunction), and this was the group that was randomized to neurohormonal blockade vs. usual care.

### Cardiac magnetic resonance imaging

All patients underwent cardiac MRI at enrolment into the study (i.e. at baseline). In the subclinical dysfunction group, MRI was repeated at the 1-year follow-up while in the overt dysfunction group, it was repeated at the diagnosis of CTRCD. No MRI results were involved in decision-making as the scans were done for research only. Magnetic resonance imaging was performed with multi-element receiver-coil array and ECG gating. Cine balanced steady-state free precession sequences were used to obtain a short axis stack of the entire ventricle for the measurement of LV volumes and LVEF. The same MRI magnet was used for both MRI studies for each patient. All MRI analyses were performed using CVI^42^ (5.9.4, Circle CVI, Calgary, Canada) in the core laboratory.

#### Intervention

Guidelines support the use of cardioprotection for patients showing a change of LVEF (i.e. meeting the overt dysfunction criteria), but there is equipoise regarding the treatment of those with isolated reduction of GLS (≥12% relative change) or sub-threshold change in EF. This equipoise justified the randomization to cardioprotection with neurohormonal antagonism or usual care at a ratio of 1:1. We used a relative change in GLS of >12% from baseline regardless of the actual strain value as this approach is consistent with prior and current guidelines A computerized protocol was used to allocate patients, with randomization in blocks of 20 at each participating centre. Cardioprotection comprised both renin–angiotensin and beta-adrenoceptor blockade. The former started with angiotensin-converting enzyme (ACE) inhibition (ramipril, target dose 10 mg daily) but in intolerant patients, this was replaced by angiotensin receptor blockers (ARBs) (e.g. irbesartan, target dose 300 mg/day, candesartan target dose 32 mg/day). Given different availability across jurisdictions, preferred beta-blockers included bisoprolol (target dose 10 mg/day) or metoprolol (target dose 100 mg/day), with a titration protocol used in the initial SUCCOUR trial (see Supplementary data online, *Appendix Table S3*).^18,19^ Doses were reported as a percentage of the target dose. Patients were assessed every 2 weeks during up-titration, to check symptom status and vital signs. In patients with side-effects, including symptomatic hypotension or bradycardia (<50/min), the dose was reduced to that prior to the last increment. Patients with intolerable side-effects of therapy could discontinue therapy but remained in the study on an intention-to-treat basis. Usual care constituted ongoing monitoring and treatment of risk factors. About one-third of patients in the usual care group were taking one (but not both) cardioprotective agents at baseline, usually at less than the target dose. These remained unchanged during the study. Both groups were followed up at 12 months.

### Endpoints

Magnetic resonance imaging images were used purely to characterize response and patients were not allocated to treatment groups based on MRI data. Analyses were performed on an intention-to-treat basis. The primary endpoint was change in 12-month MRI-defined LVEF, determined by the blinded core laboratory. The secondary endpoints at 12 months were the proportion completing planned cancer therapy, the incidence of HF, and MRI-LVEF-defined CTRCD in a categorical analysis (reduction of MRI-LVEF of >5% to <50% with symptoms of HF, or an asymptomatic reduction of MRI-LVEF of >10% to <50%).

### Statistical analysis

Sample size estimates were based on the MANTICORE-101 study of anthracyclines and trastuzumab.^20^ We anticipated a 5% reduction of LVEF in the absence of cardioprotection, with a standard deviation of ±6%. Based on a 60% risk reduction with cardioprotection,^21^ we anticipated that 70 patients per randomized group would give 80% power to identify a difference of MRI-LVEF (from 5% to 2%) and reduction of 12-month MRI-CTRCD (from 35% to 12%) with a 5% probability of Type 1 error.

Data for the screened, surveillance, and randomized groups were summarized with respect to demographic and baseline characteristics. Exploratory data analyses were performed using descriptive statistics, with standard deviation used to express variance. For the primary endpoint, between-group comparison of the change in MRI-LVEF (as a continuous variable) was assessed using a *t*-test, and analysis of covariance was used to estimate the effect size of GLS-guided cardioprotection on change of LVEF at 12 months, adjusted for baseline LVEF. Within the randomized group, the evolution of GLS compared with the previous evaluation was examined by paired *t*-tests. *T*-tests were also used for a between-group comparison of the change in GLS from the previous visit. The Fisher exact test was used to compare the infrequent secondary study endpoints, e.g. between-group incidence of CTRCD.

## Results

### Participant characteristics

The SUCCOUR-MRI trial screened 355 patients considered likely to be suitable based on anthracycline therapy and risk of CTRCD (see Supplementary data online, *Appendix Table S4*). Recruitment commenced in mid-2018, but the COVID-19 pandemic led to under-recruitment, despite an extension to August 2023.

After exclusions based upon change of planned chemotherapy, difficulties with imaging, and other reasons, 333 patients (59 ± 13 years, 79% women, Supplementary data online, *Appendix Table S4*) with normal baseline echocardiographic LVEF (>50%) but at least one other risk factor were tracked with 3-monthly echocardiography over 12 months (see Supplementary data online, *Appendix Figure S1*). Patient characteristics (*Table 1*) were consistent with patients undergoing chemotherapy at risk of CTRCD.

### Imaging findings

**Figure 1:**
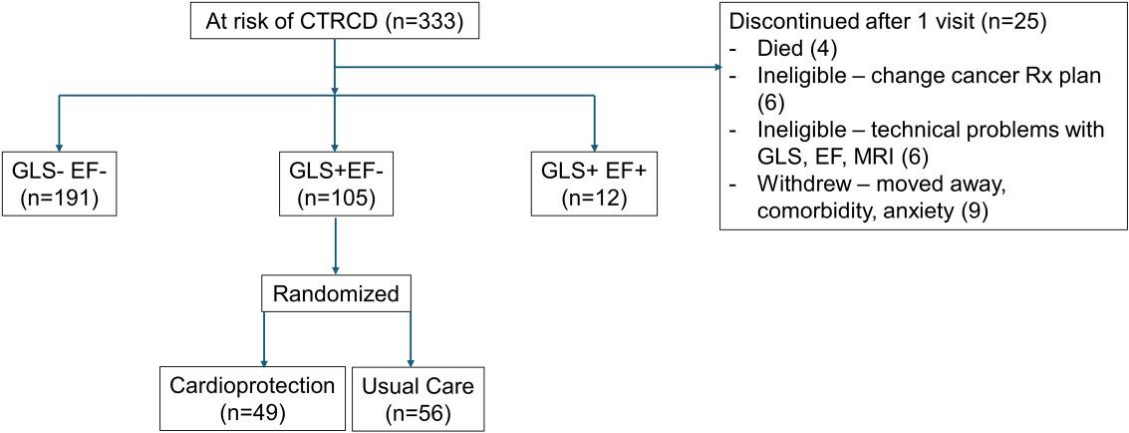
Left ventricular responses to potentially cardiotoxic chemotherapy. CTRCD, cancer-treatment-related cardiac dysfunction; CTRCD, cancer-treatment-related cardiac dysfunction; EF+, attaining threshold for CTRCD on ejection fraction criteria; EF−, not attaining threshold for CTRCD on ejection fraction criteria; GLS+, attaining threshold for CTRCD on global longitudinal strain criteria; GLS−, not attaining threshold for CTRCD on global longitudinal strain criteria

**Figure 2:**
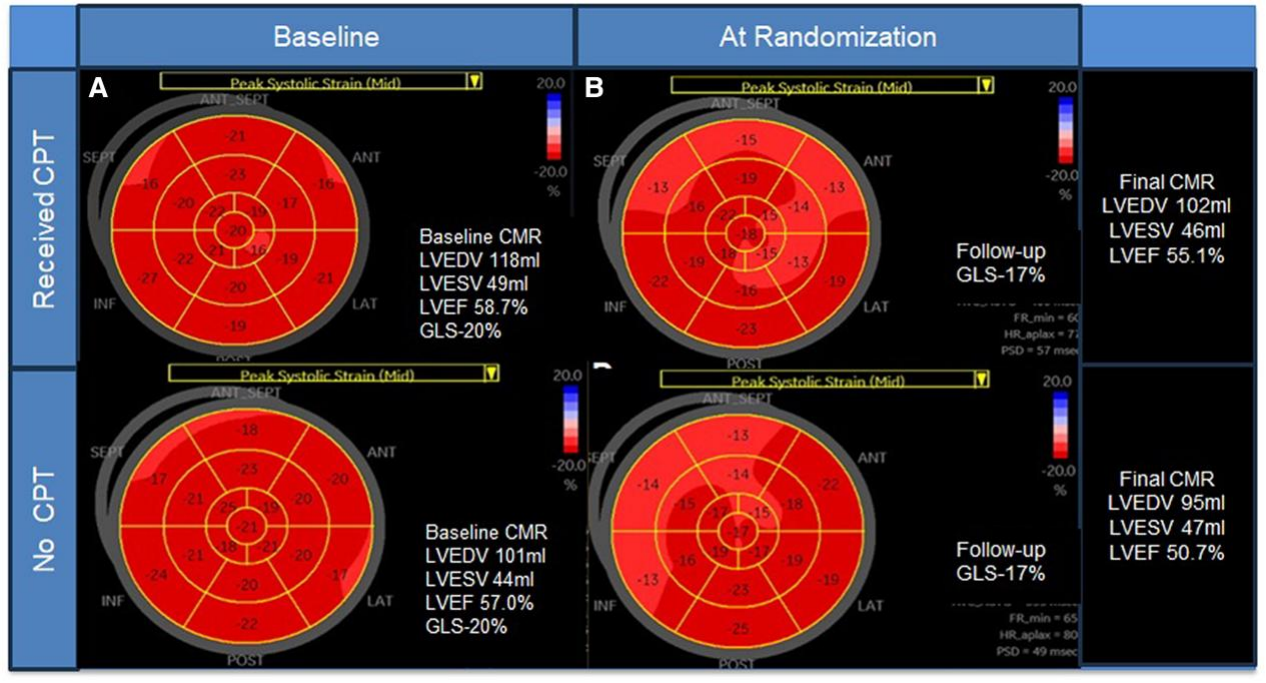
Progression of subclinical left ventricular dysfunction (impaired global longitudinal strain with preserved ejection fraction) with and without cardioprotection. The patient on cardioprotection has preserved ejection fraction at the end of follow-up, with a 3.6% drop. The patient without cardioprotection has a 6.3% deterioration of ejection fraction, almost to the abnormal range

Patients were allocated to one of three groups based on the evolution of 3-monthly echocardiograms during the trial (*Figure 1*). The largest group comprised those with no evidence of CTRCD or reduction in GLS (no CTRCD, *n* = 191), with 12 developing reduction of LVEF by >10% to <50% (LVEF-CTRCD). Patients with >12% relative reduction of GLS (*Figure 2*) in the absence of a diagnostic change of LVEF (GLS-CTRCD, *n* = 105) were similar to the overall group—75% were women, 69% had breast cancer, and their mean age was 59 ± 13 years (*Table 3*). These patients were randomized in a 1:1 ratio between cardioprotection and usual care and follow-up MRI was obtained at 12 months.

**Table 3:** Clinical features of overall screening and final randomized groups

	Cardioprotection (*n* = 49)	Usual care (*n* = 56)
Age, years	59 ± 12	58 ± 14
Female, *n* (%)	39 (80%)	40 (71%)
Heart failure factors		
Diabetes, *n* (%)	5 (10%)	7 (13%)
Hypertension, *n* (%)	14 (29%)	24 (43%)
Dyslipidemia, *n* (%)	12 (24%)	14 (25%)
Smoking^a^, *n* (%)	15 (31%)	25 (45%)
Cancer history		
Breast cancer	35 (71%)	37 (66%)
Hematologic	12 (24%)	19 (34%)
Other (sarcoma)	2 (5%)	
Cancer therapy risks		
Cumulative doxorubicin-equivalent (mg)	244 ± 48	250 ± 37
3 months or less	37 (76%)	31 (55%)
3–6 months	12 (24%)	15 (45%)
Trastuzumab, *n* (%)	19 (39%)	17 (30%)
12 months or more	18 (37%)	17 (30%)
6 months	1 (2%)	
Left chest radiation, *n* (%)	16 (33%)	19 (34%)
Left chest dose, Gy	56 ± 29	66 ± 40
Other CVD, *n* (%)^b^	7 (14%)	10 (18%)
Baseline cardioprotective therapy		
Beta blocker, *n* (%)	1 (2%)	1 (2%)
ACE inhibitor or ARB, *n* (%)	10 (20%)	19 (34%)
Statin, *n* (%)	10 (20%)	15 (27%)
Physical examination		
Systolic blood pressure, mmHg	127 ± 14	128 ± 16
Heart rate, beats/min	76 ± 13	78 ± 14
Weight, kg	78.9 ± 5.5	79.2 ± 6.0
Baseline measurement		
3D LVEF, %	62.1 ± 4.5	60.8 ± 4.1
GLS,%	−21.0 ± 1.9	−20.3 ± 2.5
MRI-LVEF %	61 ± 5	60 ± 6
Low normal MRI-LVEF (50%–53%)	5 (10%)	11 (20%)
MRI-LVEDV (mL)	147 ± 31	149 ± 31
MRI-LVESV (mL)	58 ± 16	61 ± 19

There were no significant differences between the groups.

^a^Current or prior smoking.

^b^Patients with coronary artery disease, mild valvular heart disease, and other non-major CVD (heart failure and moderate and greater valve disease excluded in both studies).

### Cardioprotection

Of the 49 participants randomized to cardioprotection, the median target dose reached of ACEi or ARB medications was 75% (inter-quartile range [IQR] 25%–100%), and for beta-blockers was 50% (IQR 25%–100%). Side-effects attributed to therapy were noted in 21 of the 49 cardioprotection patients at some stage, but in no usual care patients. Three participants were troubled by cough and had changed of an ACE inhibitor to an ARB. One had epistaxis, which continued despite dose manipulation. Dizziness attributed to low blood pressure was the most common (13 patients), and four had fatigue. These symptoms were minor—some required dose reduction but most were managed with maintaining therapy at submaximal dose. The initial systolic blood pressure fell from 126 ± 14 mmHg at baseline to 116 ± 16 mmHg (*P* = .04) at the end of the titration, with reduction throughout the six titration visits (at 2-week intervals). Likewise, the initial heart rate (76 ± 13 b.p.m.) decreased to 69 ± 11 b.p.m. (*P* = .04).

Most of the participants were randomized at 3 months (62%), with 27% at 6 months and the remainder at 9 months. Despite randomization, patients in usual care were allocated significantly later in their treatment course than patients randomized to intervention (see Supplementary data online, *Appendix Table S5*).

### Response of left ventricular ejection fraction to intervention

**Figure 3:**
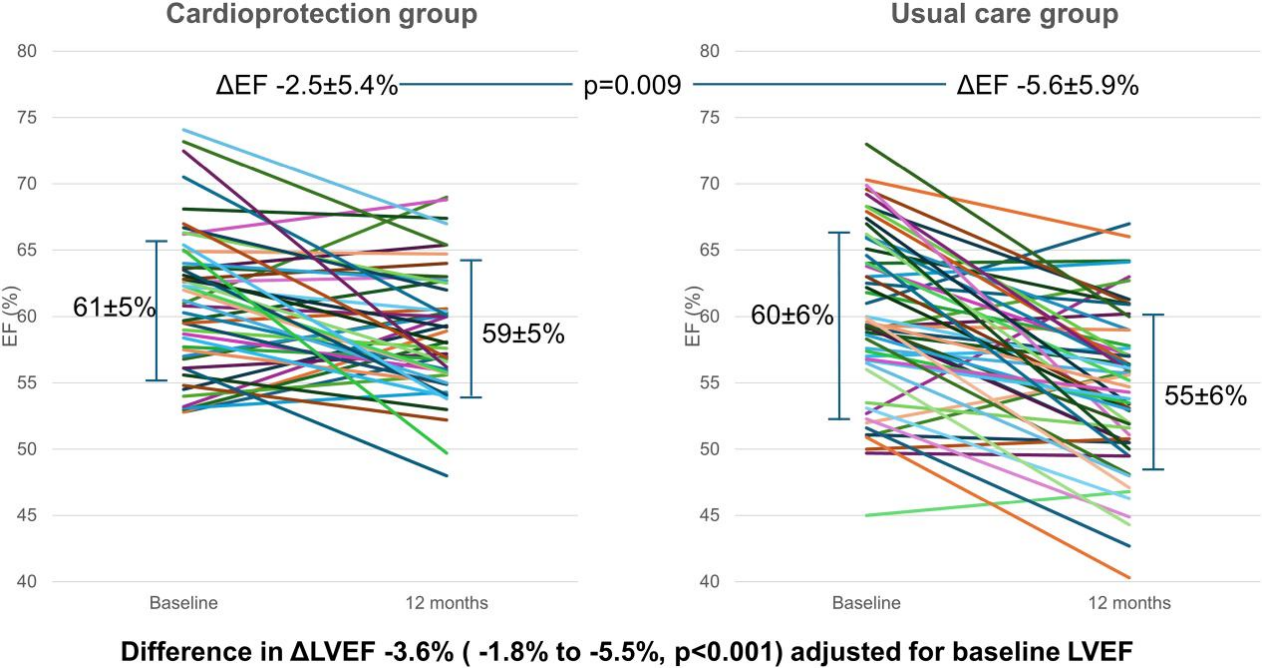
Baseline and 12-month follow-up left ventricular ejection fraction in cardioprotection and usual care groups. Baseline MRI-LVEF was similar in both groups (61 ± 5% vs. 60 ± 6%, *P* = .30), but cardioprotection was associated with better preservation of follow-up left ventricular ejection fraction than usual care. Note that study entry was based upon three-dimensional echocardiographic ejection fraction >50%, but baseline magnetic resonance imaging was ≤50% in 1 patient

During follow-up, two patients in the randomized group died (from cancer and infection) and eight (four in the cardioprotection and four in the usual care group) were unable to undertake the 12-month MRI—in these participants, the most recent (generally, 9 months) 3D LVEF was substituted. *Figure 3* shows the similar baseline MRI-LVEF in both groups (61 ± 5% vs. 60 ± 6%, *P* = .30), with a similar proportion of patients in the usual care group who had a low normal EF (50%–55%). There was better preserved mean LVEF at follow-up in the cardioprotection group rather than usual care (59 ± 5% vs. 55 ± 6%, *P* < .0001). The change in LVEF from baseline to 12 months was −2.5 ± 5.4% in the cardioprotection group and −5.6 ± 5.9% in the usual care group (*P* = .009). In analysis of covariance, adjusted for baseline LVEF, GLS-guided cardioprotection led to a 3.6% reduction (95% CI −5.5% to −1.8%, *P* < .001) in the decrement of LVEF. A post-hoc comparison of the outcomes if patients were randomized at a 15% (vs. a 12%) change in GLS provided similar results (see Supplementary data online, *Appendix Table S6*).

The mean difference between 3D- and MRI-LVEF at baseline was 1 ± 6%, and at 12 months was 1 ± 3%. While there was a difference in LVEF between the two groups when 3D rather than MRI results were used in all patients, this was non-significant (−2.4 ± 3.7 vs. −4.0 ± 4.7%, *P* = .08). Exclusion of patients without 12-month MRI-LVEF provided similar findings to the primary analysis in the change in MRI-LVEF (−2.6 ± 5.3 vs. −6.3 ± 5.6%, *P* = .001).

### Secondary endpoints

No patients discontinued cancer therapy due to HF. No patients in the cardioprotection arm and two patients in the usual care arm developed HF (*P* = .49). At 12 months, CTRCD developed in 1/49 in the cardioprotection group, compared to 6/56 (five asymptomatic and one symptomatic) in the usual care group (*P* = .075).

### Global longitudinal strain changes

**Figure 4:**
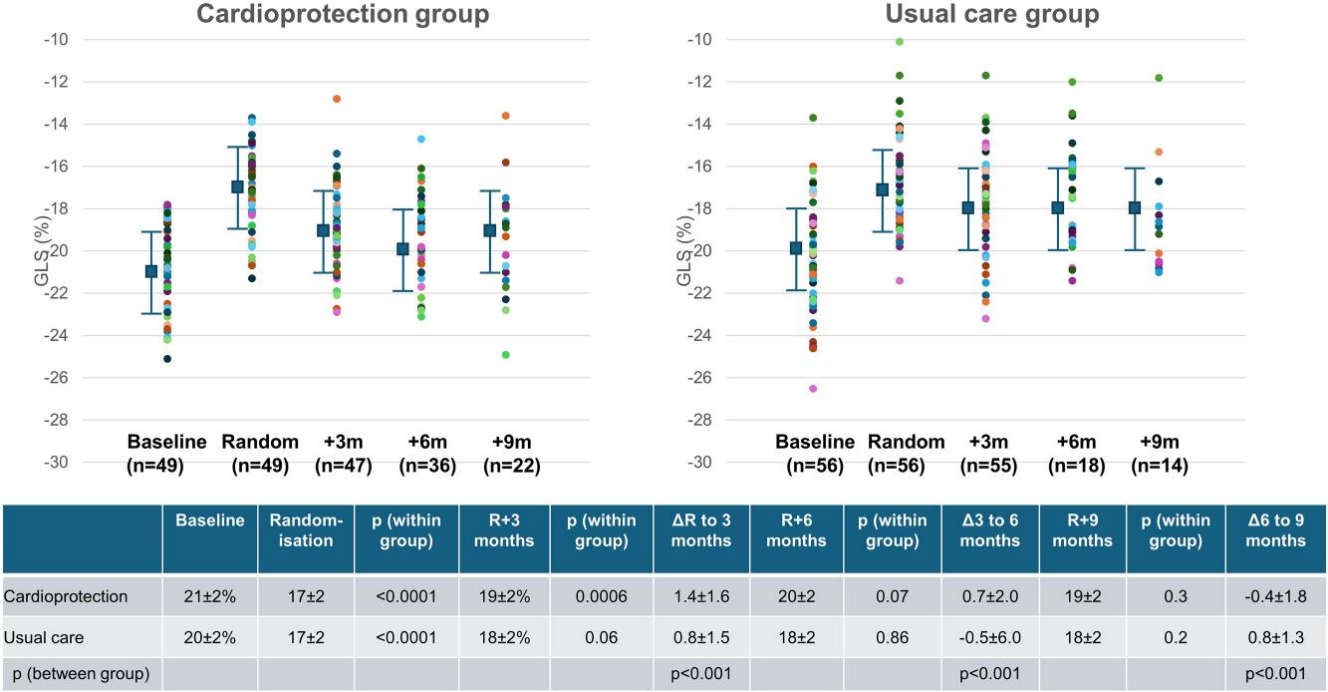
Evolution of global longitudinal strain in patients randomized to cardioprotection and usual care. Individual data-point, mean and standard deviation show that the reduction in global longitudinal strain between the start of echo surveillance (baseline) and the time of randomization was similar in both groups. However, the trajectory after 3, 6, and 9 months post-randomization (R + 3, R + 6, R + 9) was different, with recovery of global longitudinal strain in the cardioprotection group, and minimal change in the usual care group. The accompanying table shows within and between-group differences, as well as numbers of patients. Data are sparse at 9 months post-randomization (22 in cardioprotection and 14 in usual care). The *P* values compare the current visit vs. the prior visit

The deterioration of GLS from baseline to randomization was of similar degree in the cardioprotection (21 ± 2% to 17 ± 2%, *P* < .0001) and usual care groups (20 ± 2% to 17 ± 2%, *P* < .0001). *Figure 4* shows the evolution of GLS in patients after randomization and compares measurement at each time-point. In the cardioprotection group, there was an improvement at 3 months after randomization (19 ± 2%, *P* = .0006). However, in the usual care group, GLS was similar at 3 months (18 ± 2%, *P* = .06) and 6 months (18 ± 2%, *P* = .86).

### Reproducibility

In 17 randomly selected images, intra-observer mean differences for MRI-EDV, -ESV, and -EF were, respectively, .2 mL (95% confidence interval [CI] −4.1 to 4.5 mL), −.8 mL (−6.3 to 4.6 mL), and .6% (−2.7–3.9). Inter-observer mean differences for MRI-EDV, -ESV, and -EF were respectively .7 mL (95% CI −5.6 to 6.9 mL), 3.0 mL (−1.9 to 7.9 mL), and −1.8% (−5.0 to 1.4%), with respective percentage differences of .9% (−3.9 to 5.6%), 5.0% (−3.1 to 13.2%), and −3.3% (−8.8 to 2.3%). The mean (95% CI) difference in GLS between the sites and the core laboratory was −.44 (−1.51 to .64%).

## Discussion

The SUCCOUR-MRI trial showed that, in patients with reduced GLS during surveillance in the absence of accepted LVEF criteria, cardioprotective therapy was associated with better preservation of 12-month MRI-LVEF compared with usual care (*Structured Graphical Abstract*). The categorical diagnosis of CTRCD was also less common after cardioprotective therapy although the trial had limited power to assess whether cardioprotection has an impact on this secondary outcome. We hope that these findings clarify some uncertainty about the management of patients with isolated reduction of GLS, reflected in the Class IIa indication in the current cardio-oncology guidelines.^6^

### Imaging-targeted cardioprotection

There are multiple causes of LV dysfunction and HF in cancer survivors, but CTRCD is an important component. Several prevention strategies have been proposed. Avoidance of potentially cardiotoxic drugs is always desirable, but often not possible—e.g. anthracyclines remain indispensable in the treatment of many haematologic malignancies, sarcomas, and some breast cancers.^1^ Even if anthracycline-related risks were contained by wider use of dexrazoxane,^22^ the problem of CTRCD would remain a concern, due to the development of non-anthracycline therapies—principally monoclonal antibodies and tyrosine kinase inhibitors—that are also cardiotoxic.^2^

Cardioprotective strategies may be effective in the prevention of CTRCD, with the strongest evidence for neurohormonal inhibition^23^ and less definite benefit from statins.^24^ However, there is insufficient evidence to justify a uniform prophylactic approach to all patients taking potentially cardiotoxic therapies—because at least 80% will never develop cardiotoxicity, and at least 20% (more in this trial) will develop side-effects of dizziness and hypotension in response to neurohormonal inhibition during chemotherapy.^25^ A targeted approach to prevention, based on imaging and/or biomarker surveillance followed by treatment of subclinical dysfunction, is therefore the principal practice strategy.^26^ Despite the attraction of using biomarkers (e.g. B-type natriuretic peptide, troponin) instead of imaging for surveillance, this strategy is not in uniform use, possibly reflecting the heterogeneity of reports about its efficacy, perhaps reflecting the susceptibility of natriuretic peptides to loading conditions and renal impairment. The weaknesses of this approach are that screening techniques need to be sufficiently accurate to identify at-risk patients, and that ongoing surveillance is important, as the response rate progressively decreases with increasing delay between the onset of CTRCD and the initiation of cardioprotection in patients receiving anthracyclines.^27^

The measurement of LVEF is a unifying feature of guideline-supported screening-directed strategies.^6^ While the management of patients with a significant reduction of LVEF is well-accepted,^6^ most patients developing CTRCD do not show a significant reduction of LVEF during chemotherapy.^28^ Part of the explanation for this may be that the limited test–retest reproducibility of echocardiographic LVEF has led to CTRCD criteria based on substantial impairment (10% absolute change) of LVEF, which is insufficiently sensitive to minor change.

### Previous evidence for global longitudinal strain

Global longitudinal strain is a marker of LV systolic shortening that has been in use over the last two decades.^29^ Longitudinal shortening is thought to largely represent subendocardial function, and as the subendocardium is sensitive to injury, GLS is a sensitive marker of early myocardial disease. This test has a high level of feasibility, but is susceptible to image quality (e.g. compromised by difficult echo windows after left mastectomy or radiation) and haemodynamic influences (changes in blood pressure or volume load during chemotherapy). Due to differences in the default approach to measuring GLS between different software providers, sequential GLS measurements need to be assessed with the same software.

A number of observational studies have shown that patients with a 10%–15% relative change of GLS (i.e. a 2%–3% absolute change) are at risk of developing CTRCD.^11^ These studies show that GLS may show a meaningful change among patients showing a <10% change of LVEF, or a change within the normal range. Despite these observational data, clinical trial confirmation of the value of GLS-guided cardioprotection has been sparse.

The initial SUCCOUR trial randomized patients at risk of CTRCD to a GLS-guided or an LVEF-guided strategy to initiate cardioprotection.^30^ That study showed no difference in the change of LVEF (primary outcome) in the GLS-guided vs. LVEF-guided cardioprotection patients at 1 and 3 years of follow-up. Although fewer GLS-guided patients developed CTRCD, this was statistically significant at 1 but not 3 years.^19^ There were three important limitations to the original SUCCOUR trial. First, although the participants had a range of risks, the average LVEF reduction was 3% by 3D echocardiography—less than the reproducibility of the measurement. This alone may have explained the results, especially as echocardiographic LVEF measurements lack precision. Second, only 64 patients (44 of GLS-guided and 20 of the LVEF-guided patients) were eligible for cardioprotection, and only 37 of these patients were treated for an isolated reduction of GLS. Third, although 3D echocardiography was used for measurement of the reference standard, due to image quality limitations 2D echocardiography had to be used in a subgroup of patients, with consequent concern that changes in LVEF may have been underestimated. The SUCCOUR-MRI trial has some important differences from the original SUCCOUR study design,^30^ including randomization of patients with an isolated GLS change to cardioprotection vs. standard of care, the use of MRI-based LVEF for the primary outcome measure, and a higher risk level of this group compared to the original SUCCOUR trial (*Table 1*). This latter point is important with respect to the place of imaging surveillance, which can be difficult to resource and organize. Nonetheless, 6/56 (11%) of the usual care group developed CTRCD at 12 months. The challenge here is the calculation of clinical risk, which remains difficult.

The criteria for CTRCD have evolved over time. In the original SUCCOUR trial, this was identified as a fall to EF <55% (as was the norm at the time), with CTRCD being identified in 5.8% of GLS-guided and 13.7% of the EF-guided groups. This is different from SUCCOUR-MRI, which used the 50% cutoff used in the current CTRCD criteria. Had we used the 55% criteria in SUCCOUR-MRI, there would have been 10 patients (18%) with CTRCD in the usual care and 2 in the 49 (4%) in the cardioprotection group (*P* = .03).

### Limitations

The SUCCOUR-MRI trial has several limitations. Most importantly, the goal of cardioprotection is to reduce HF, but this is uncommon and generally occurs in late follow-up. Therefore, this, like all other trials in this field, uses follow-up LVEF as a surrogate of HF risk. While there was worse LVEF impairment in the absence of cardioprotection, most patients had LVEF measurements within the normal range. The usual care group had more patients with a borderline baseline LVEF, who deteriorated over 12 months.

Second, it was initially intended to randomize 140 patients, but the interruptions caused by the COVID-19 pandemic led to the conclusion of the trial after 105 were randomized. Although the resulting reduction of statistical power did not prevent showing a significant benefit in favour of cardioprotection, the confidence intervals of the effect size are larger than they might otherwise have been, and differences in CTRCD between arms at 12 months did not reach statistical significance. Nonetheless, a meaningful reduction of LVEF to below the normal range by MRI (i.e. LVEF < 50%) was almost exclusive to the group not provided cardioprotection. Third, as is inevitable in trial at the time of chemotherapy, some patients died and others were unable to undertake the final MRI test, although we designed 3D echocardiography follow-up in anticipation of this problem, and 3D echocardiography and MRI-LVEF correlated well, with median differences from 1% and 3% at baseline and follow-up.

Fourth, despite randomization, more patients with early change of GLS were allocated to cardioprotection, and more patients with late changes of GLS were allocated to usual care (see Supplementary data online, *Appendix Table S4*). As patients were followed over a year from the start of imaging surveillance, people developing change late may not have had time to recover. On the other hand, *Figure 4* shows little recovery of GLS at any stage in the control group, and rapid recovery in the treatment group.

Fifth, the GLS criteria used in this study differ from the current guideline cutoff of 15%, reflecting the fact that the study was designed well before the guidelines were launched in 2022. In fact, the 15% cutoff in the guidelines, based on expert opinion, derives from a paper in which the best threshold to identify CTRCD risk (in a population similar to that studied) was 11% relative change, with an upper 95% CI of 14.6%. A post-hoc sub-analysis using a 15% change (see Supplementary data online, *Appendix Table S6*) showed little impact from the different thresholds—in the usual care group, the 15% cutoff would have led to treatment of fewer patients but missed two with CTRCD. As GLS change >12% was used to initiate randomization, there are problems with trying to fully deconvolute this with a different cutoff.

Sixth, the study was confined to imaging rather than biomarkers. Finally, the trial was limited to neurohormonal inhibition (which is the most widely used form of cardioprotection) and did not include other potentially protective agents such as statins, sacubitril/valsartan, and sodium-glucose cotransporter-2 inhibitors.

## Conclusions

The SUCCOUR-MRI trial demonstrated that approximately one-third of patients deemed at risk of CTRCD developed an isolated reduction in GLS. A cardioprotection strategy based on the renin–angiotensin system and beta-adrenoceptor inhibition resulted in better preservation of 12-month MRI-LVEF compared with usual care. These results support the value of GLS, even in the absence of threshold changes of LVEF in the follow-up of patients at risk of CTRCD.

## Supplementary data

Supplementary data are available at *European Heart Journal* online.

## Declarations

### Disclosure of Interest

Nothing to declare.

## Supplementary Material

ehae574_Supplementary_Data

## Data Availability

The data underlying this article will be shared on reasonable request to the corresponding author.

## Pre-registered Clinical Trial Number

Australian New Zealand Clinical Trials Registry (ACTRN12620001094965).
