# Supplementary material for: Strain surveillance during chemotherapy to improve cardiovascular outcomes: the SUCCOUR-MRI trial
Source: Eur Heart J. 2024 Sep 1;45(41):4414–24. doi: 10.1093/eurheartj/ehae574 (PMC11542702; doi:10.1093/eurheartj/ehae574)

**Supplementary material**

# Strain surveillance during chemotherapy to improve cardiovascular outcomes: the SUCCOUR-MRI trial

**Appendix Table 1. SUCCOUR-MRI sites, recruited patients and investigators**

| Country | Center Name | Site Investigators |
|---|---|---|
| **Australia** | Royal Adelaide Hospital, Adelaide (65) | Mitra Shirazi, Julie Souness |
| | Westmead Hospital, Sydney (62) | Liza Thomas, Luke Stefani |
| | Advara Healthcare, Perth (58) | Peter Dias, Hayley Mammatt |
| | Baker Heart and Diabetes Institute, Melbourne (46) | Mark Nolan, Elizabeth Dewar |
| | Flinders Medical Centre, Adelaide (17) | Joseph Selvanayagam, Sau Lee |
| | Advara Healthcare, Melbourne (16) | Leighton Kearney, Piyush Srivastava |
| | Advara Healthcare, Brisbane (14) | Ben Fitzgerald |
| | Royal Brisbane and Women's Hospital, Brisbane (12) | John Atherton, Leeanne Palethorpe |
| | Nepean Hospital, Sydney (10) | Kazuaki Negishi, Tomoko Negishi, Christopher Yu |
| | Calvary Mater Hospital, Newcastle (8) | Aaron Sverdlov, Naomi Knoblauch |
| | Princess Alexandra Hospital, Brisbane (1) | Sudhir Wahi, Cindy Hall |
| | Liverpool (1) | James Otton |
| **Canada** | Peter Munk Cardiac Center, Toronto General Hospital, University Health Network, Toronto (44) | Paaladinesh Thavendiranathan, Eitan Amir, Judy Kim |

**Appendix Table 2. Study procedures.** The randomisation time-point is dependent on the time of a 12% reduction of GLS, and could occur at 3, 6, or 9 months.

| Study Procedures | Screening/ Baseline | Month 3 | Month 6 | Month 9 | Month 12 | Early Discontinuation Visit | 12 Months After Early Dis-continuation Visit |
|---|---|---|---|---|---|---|---|
| Informed Consent | X | | | | | | |
| Medical History [a] | X | | | | | | X |
| Physical Exam | X | | | | | X | |
| 12 Lead ECG (with arrhythmia assessment, conduction abnormalities)[b] | X | | | | | X | |
| Echocardiogram | X | X | X | X | X | | |
| MRI | X | | | | X* | X† | |
| Questionnaire (EQ-5D-5L) | X | X | X | X | X | | X |
| Vital Signs (BP, HR, RR) | X | X | X | X | X | | |
| Usual care labs | X | X | X | X | X | | |
| Concomitant Medications Review | X | X | X | X | X | | X |
| AE/SAE Assessment | X | X | X | X | X | | X |
| Heart Failure Assessment (NYHA-HF) | X | X | X | X | X | | X |
| Chemotherapy Regimen & Review [c] | X | X | X | X | X | | X |
| Review of dispensing / continuing cardioprotective drug [d] | | X | X | X | X | | |

a- History of cardiovascular risk factors, known coronary artery disease, valvular heart disease; body mass index, side of breast cancer, history of diabetes mellitus, history of smoking, total cholesterol

b- 12 lead ECG

c- Time from chemotherapy completion to HF symptoms; Interruptions in planned chemotherapy; completion or discontinuation of chemotherapy, cancer therapy doses.

d- Patients who have strain evidence of cardiotoxicity were randomised to cardio-protective therapy

X* - MRI completed at month 12 for patients randomised to cardioprotection

†Refers to MRI performed at the time of LVEF defined CTRCD in the group of patients who developed CTRCD without transitioning through a significant change in GLS.

**Appendix Table 3. Sample medication management in cardioprotection and usual care groups**

| **Patient group** | **Medications** | **Week since** randomisation | | | | | | | |
|---|---|---|---|---|---|---|---|---|---|
| **Usual care** | | 0 | 2 | 4 | 6 | 8 | 10 | 12 | 14 |
| Treatment naive | Nil | No treatment | | | | | | | |
| Baseline therapy | BB only | Maintain BB dose | | | | | | | |
| | ACEi/ARB only | Maintain ACEi/ARB dose | | | | | | | |

| **Cardioprotection** | **Medications** | **0** | **2** | **4** | **6** | **8** | **10** | **12** | **14** |
|---|---|---|---|---|---|---|---|---|---|
| Treatment naive | Ramipril (mg/d) | 2.5 | 5.0 | 5.0 | 5.0 | 5.0 | 10 | 10 | 10 |
| | Bisoprolol (mg/d) | | | 1.25 | 2.5 | 5.0 | 10 | 10 | 10 |
| Baseline BB (eg 5mg/d) | Ramipril (mg/d) | 2.5 | 5.0 | 10 | 10 | 10 | 10 | 10 | 10 |
| | Bisoprolol (mg/d) | 5.0 | 5.0 | 10 | 10 | 10 | 10 | 10 | 10 |
| Baseline ACEi (eg 5mg/d) | Ramipril (mg/d) | 5.0 | 5 | 5.0 | 10 | 10 | 10 | 10 | 10 |
| | Bisoprolol (mg/d) | 1.25 | 2.5 | 5.0 | 10 | 10 | 10 | 10 | 10 |

**Appendix Table 4. Clinical and imaging features of screened, follow-up and randomised patients**

| | **Screened (n=355)** | **Follow-up group (n=333)** | **Randomised (n=105)** |
|---|---|---|---|
| Age, years | 60±13 | 59±13 | 59±13 |
| Female, n (%) | 283 (80%) | 262 (79%) | 79 (75%) |
| Heart failure factors | | | |
| Diabetes, n (%) | 59 (17%) | 55 (17%) | 12 (11%) |
| Hypertension, n (%) | 133 (38%) | 128 (38%) | 38 (36%) |
| Dyslipidemia, n (%) | 103 (29%) | 102 (31%) | 26 (25%) |
| Smoking*, n (%) | 116 (33%) | 125 (38%) | 40 (38%) |
| Cancer history; | | | |
| Breast cancer | 260 (73%) | 251 (75%) | 72 (69%) |
| Hematologic | 84 (24%) | 77 (23%) | 31 (30%) |
| Other (sarcoma) | 11(3%) | 2 (1%) | 2 (2%) |
| Other CVD, n (%)[#] | 53 (15%) | 52 (16%) | 17 (16%) |
| Baseline cardioprotective therapy | | | |
| Beta blocker, n (%) | 13 (4%) | 11 (3%) | 1 (2%) |
| ACE inhibitor or ARB, n (%) | 91 (26%) | 89 (27%) | 10 (20%) |
| Statin, n (%) | 99 (30%) | 96 (29%) | 10 (20%) |
| Physical examination | | | |
| Systolic blood pressure, mmHg | 127±15 | 128±16 | 128±15 |
| Heart rate, beats/min | 77±14 | 75±10 | 77±14 |
| Weight, kg | 79.1±5.8 | 77±14 | 79.0±5.8 |
| Baseline measurement | | | |
| 3D LVEF, % | 61.4±4.3 | 61±6 | 62.1±4.5 |
| GLS,% | -20.6±2.3 | -19.8±2.5 | -21.0±1.9 |
| MRI-LVEF % | 62±9 | 62±9 | 61±5 |

**Appendix Table 5.** Timing of development of subclinical LV dysfunction in cardioprotection and usual care groups

| | 3m | 6m | 9m | Total |
|---|---|---|---|---|
| **CPT** | 31 | 13 | 5 | 49 |
| **UC** | 19 | 22 | 15 | 56 |
| | 50 | 35 | 20 | 105 |

**Appendix Table 6. Change in 12-month EF according to the timing and magnitude of GLS change in the cardioprotection and usual care groups.** The results appear similar with the 12% and 15% cutoffs. In the usual care group, the 15% cutoff would have led to treatment of 69 rather than 105 patients but missed 2 with CTRCD. The change of EF was similar between different cutoffs.

| | **Delta GLS** | **Total** | **3m** | | **6m** | | **9m** | | **CTRCD (n)** |
|---|---|---|---|---|---|---|---|---|---|
| | | | **n** | **ΔEF** | **n** | **ΔEF** | **n** | **ΔEF** | |
| **Cardio-protection** | >12% | 49 | 31 | -3.6±5.2 | 13 | -3.0±7.0 | 5 | -0.7±3.0 | 1 |
| | >15% | 30 | 19 | -3.8±6.0 | 8 | -2.0±5.7 | 3 | -1.0±1.9 | 1 |
| **Usual care** | >12% | 56 | 19 | -6.5±6.4 | 22 | -5.3±6.0 | 15 | -6.4±5.3 | 6 |
| | >15% | 39 | 11 | -6.6±6.8 | 18 | -4.8±5.3 | 10 | -9.0±5.1 | 4 |

**Appendix Figure 1. Imaging surveillance after potentially cardiotoxic chemotherapy.** All eligible patients underwent baseline echocardiography and cardiac magnetic resonance (CMR) at the time of recruitment, and follow-up echocardiography at 3-month intervals. Randomised patients underwent 12-month CMR and change of LVEF from baseline to 12 months was the primary outcome. GLS, global longitudinal strain, M=months, EF=ejection fraction.

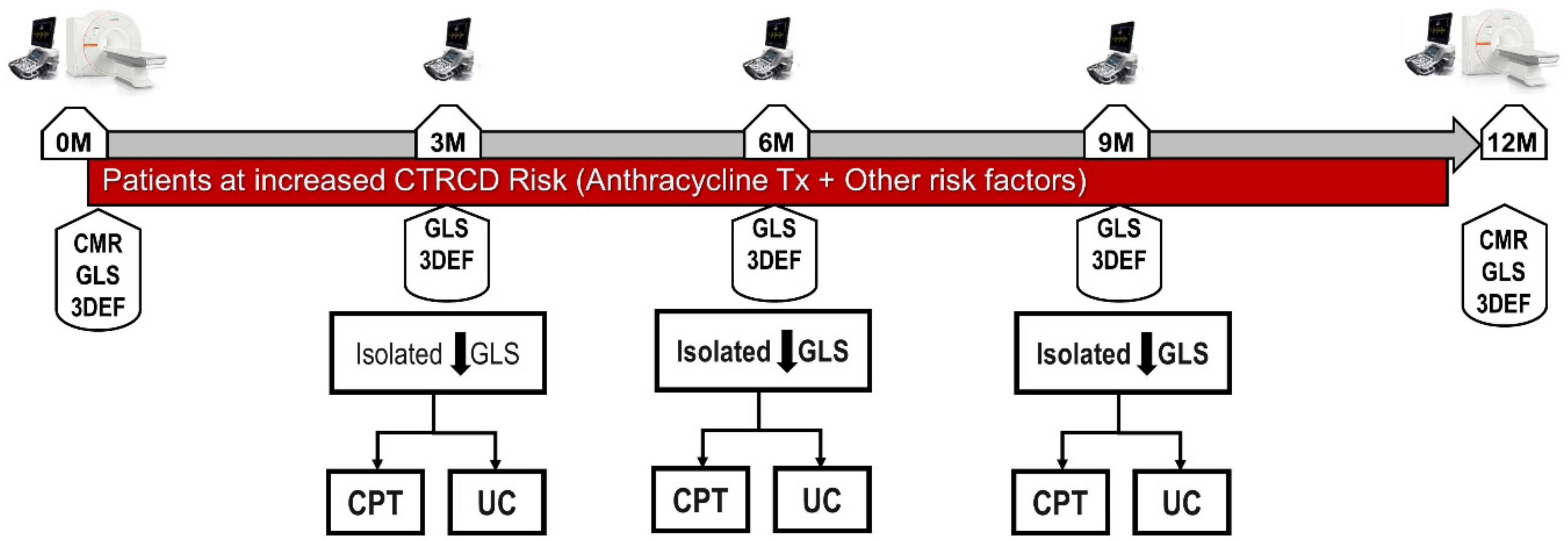

Supplement: ehae574_Supplementary_Data [file ehae574_supplementary_data.pdf]
